# An Overview of Seed Germination Mechanisms as a Target for Seed Priming

**DOI:** 10.3390/ijms27209250

**Published:** 2026-10-18

**Authors:** Galina Smolikova, Vadim Demidchik, Sergei Medvedev

**Affiliations:** 1 Department of Plant Physiology and Biochemistry, St. Petersburg State University, 199034 St. Petersburg, Russia; g.smolikova@spbu.ru; 2 V.F. Kuprevich Institute of Experimental Botany of the National Academy of Sciences of Belarus, 220072 Minsk, Belarus; demidchik@botany.by

**Keywords:** higher plants, plant development, seed germination, seed priming, hydropriming, stress resilience, sustainable agriculture

## Abstract

As a central developmental phenomenon, seed germination is highly vulnerable to abiotic stresses, which strongly influence subsequent plant development and ultimately determine crop establishment and yield. An improved understanding of seed germination mechanisms has enabled the development of diverse crop enhancement strategies. Among these, seed priming has become a key tool in sustainable agriculture, widely used to boost plant stress tolerance and improve overall productivity. Among the various priming techniques, hydropriming involves the controlled hydration of seeds that initiates germinative events, followed by drying to arrest metabolism. The effectiveness of hydropriming depends on the precise modulation of germination to activate stress-related physiological responses. However, limited knowledge of the molecular dynamics underlying seed germination remains the main bottleneck for developing next-generation priming methods. This review comprehensively covers the genetics, epigenetics, and biochemical mechanisms of germination induced by hydropriming of seeds in higher plants.

## 1. Introduction

Germination is initiated when seeds are exposed to appropriate environmental conditions, which are species-specific and include optimal levels of humidity, temperature, and light [1,2,3]. As a critical yet vulnerable phase in the plant life cycle, seed germination is frequently disrupted by environmental and biological stresses, triggering erratic seedling emergence. Advances in our understanding of the mechanisms governing seed germination have facilitated the emergence of ‘priming’ techniques which are aimed at improving seedling performance. In general, the concept of priming is based on the natural plant strategies of stress adaptation. While some of these strategies are constitutive, others are induced only in response to stress [4,5]. The main goal of seed priming is to enhance seed germination and seedling resistance to subsequent stress. Various techniques are classified into hydropriming, osmopriming, hormopriming, halopriming, solid matrix priming, nutrient priming, biopriming, nanopriming, magnetopriming, ultraviolet priming, gamma radiation priming, cold plasma priming, electron priming, and laser priming and others [6,7,8,9,10,11,12,13,14,15,16,17,18,19,20,21,22]. Notably, this techniques are effective for bolstering plant tolerance to drought [9,23,24], heat [25], and salinity [7,26].

Among different types of priming, hydropriming requires no chemicals and eliminates the risk of soil or crop contamination. It involves the controlled water uptake to seeds to activate metabolic pathways without triggering radicle protrusion, followed by redrying to the initial water content [9,10,23,24,27]. In this way, hydropriming mimics the natural wet–dry cycles that seeds encounter during spring soil droughts and subsequent rehydration [28]. Consequently, seeds originating from plants in climatically harsh regions have evolved inherent tolerance to fluctuating soil moisture [10,12,29,30].

According to the Scopus database, research on seed priming has experienced a remarkable surge, with approximately 2000 to 2500 articles published over the past five years and more than 600 papers appearing in 2025 [5]. However, alongside the growth in publication activity, evidence is accumulating for the significant dependency of priming on genotype, stress intensity, and even seed quality. Despite well-documented practical implementation, effects of priming remain largely empirical [31]. The molecular dynamics of priming, such as the temporal sequence of DNA repair activation, antioxidant systems, storage reserve mobilization, and hormonal signaling, remain incompletely characterized. While the transgenerational effects of priming are widely studied, the idea that induced tolerance persists in subsequent generations through epigenetic mechanisms remains a working hypothesis. In this review, we synthesize the current knowledge of seed priming for stress resilience, with a particular focus on hydropriming and germination metabolism.

## 2. Metabolic Processes in Germinating Seeds as Potential Targets for Priming

**Figure 1:**
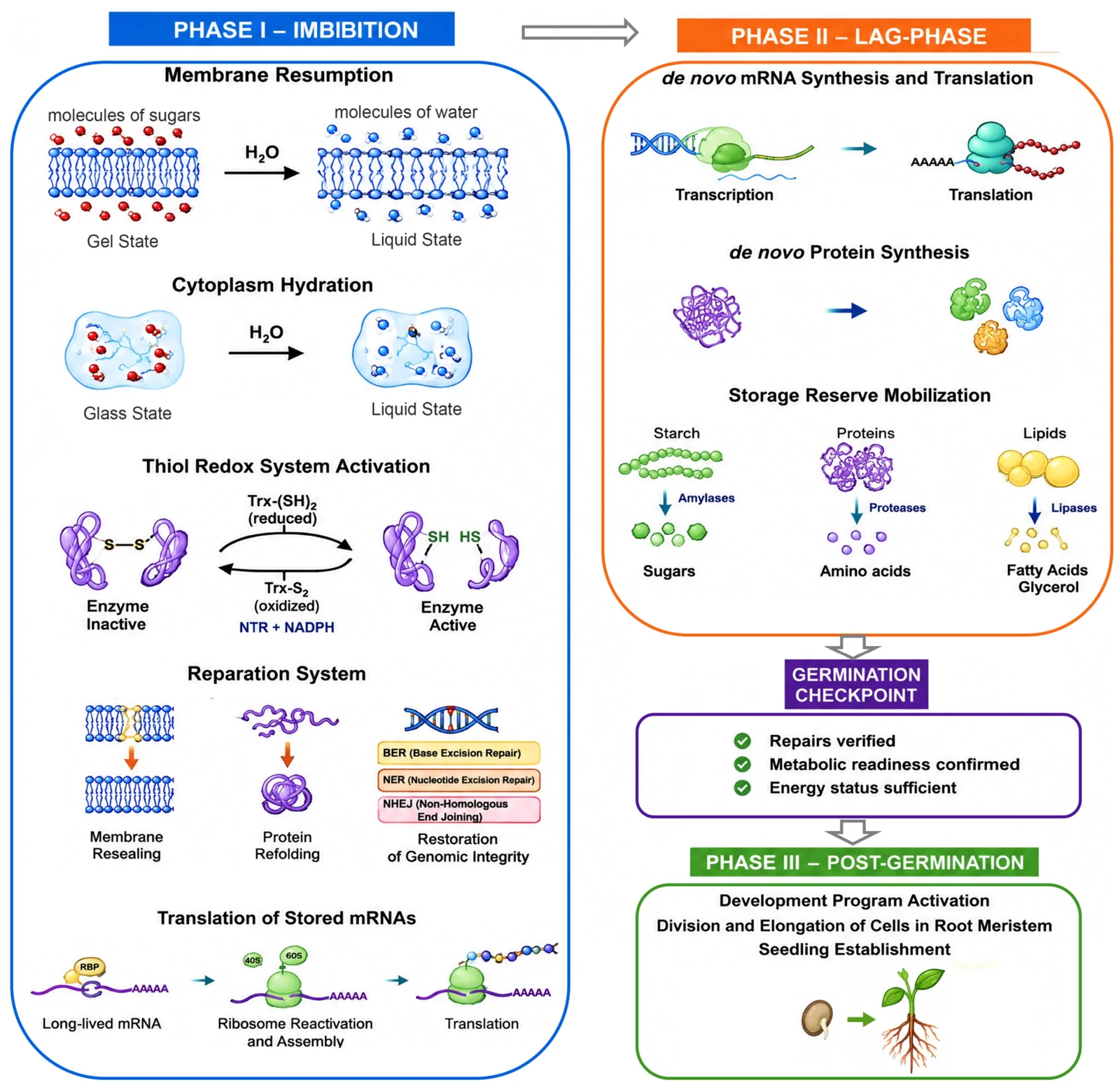
**Metabolic processes occurring during seed germination, organized into three phases**. **Phase I—Imbibition** (*blue panel*): Upon water uptake, the membranes transit from a gel to a liquid state, and the cytoplasm shifts from a glassy to a liquid state. The thiol-redox system is activated when NADPH-dependent thioredoxin reductase (NTR) reduces the oxidized form thioredoxin (Trx-S_2_) to its reduced form (Trx-SH_2_), which subsequently converts inactive enzymes into their active forms. The repair system reseals membranes, refolds proteins, and restores genomic integrity through base excision repair (BER), nucleotide excision repair (NER), and non-homologous end-joining (NHEJ) pathways. Concurrently, stored long-lived mRNAs bound to RNA-binding proteins (RBPs) are translated following ribosome reactivation and assembly. **Phase II—Lag-phase** (*orange panel*): De novo mRNA synthesis and translation, following by protein turnover, take place. Storage reserve mobilization initiates converting starch, proteins, and lipids into sugars, amino acids, and fatty acids, respectively. **Germination checkpoint (***purple panel*): Before committing germination, the seed verifies DNA repair, confirms metabolic readiness, and ensures sufficient energy status. **Phase III—Post-germination** (*green panel*): Developmental program activation leads to the division and elongation of cells in the root meristem, culminating in seedling establishment.

Seed germination begins with water uptake, ends with the embryonic root emergence through the seed coat, and is typically divided into three phases [1,31,32] (Figure 1). Phase I (imbibition) involves activation of water uptake driven by a very negative matric potential (approximately −150 MPa), leading to hydration of macromolecules, coat softening, and respiration activation. This phase also initiates damage repair of macromolecules, membranes, and cell organelles including mitochondria [1,32,33,34,35]. In Phase II (also known as the lag phase), the water potential increases to approximately −1.5 MPa, primarily due to its osmotic component, and the rate of water uptake decreases, reaching a plateau. The main metabolic processes of germination are activated in this phase: mobilization of reserve substances, germination-specific protein synthesis (on both stored and newly synthesized mRNA templates), and their post-translational modifications (PTM) [3,32,36]. Activation of respiratory metabolism results in the dramatic up-regulation of the production of reactive oxygen species (ROS), triggering the synthesis of antioxidants, such as enzymatic and low-molecular-weight compounds [37,38]. The end of Phase II is usually defined as the moment of radicle protrusion. The beginning of Phase III, also called post-germination, marks the initiation of embryonic root growth [31,39]. The seed-to-seedling transition, marked by the initiation of embryonic root growth, represents a pivotal juncture in the plant life cycle. Notably, ‘orthodox’ seeds retain desiccation tolerance throughout germination phases I and II, during which the process remains fully reversible [40,41,42]. This reversible interval, which incurs no loss of germination capability, is commonly termed the “*window of desiccation tolerance*” [40]. In this context, the transition from phase II to phase III is a critical moment in plant ontogenesis [42,43,44,45].

Seed priming is mostly based on hydration–dehydration cycles of germinating seeds, which involve controlled imbibition either in water (hydropriming) or in osmotic solutions (osmopriming), followed by dehydration [18,24,25,26,27,28,29,30,31]. In nature, plants exposed to alternating desiccation and rehydration cycles effectively undergo this same priming process naturally. This regulated hydration allows seeds to progress through the initial phases of germination but deliberately halts them before radicle emergence, ensuring that the seed coat remains intact, and that the process remains reversible [46,47].

**Figure 2:**
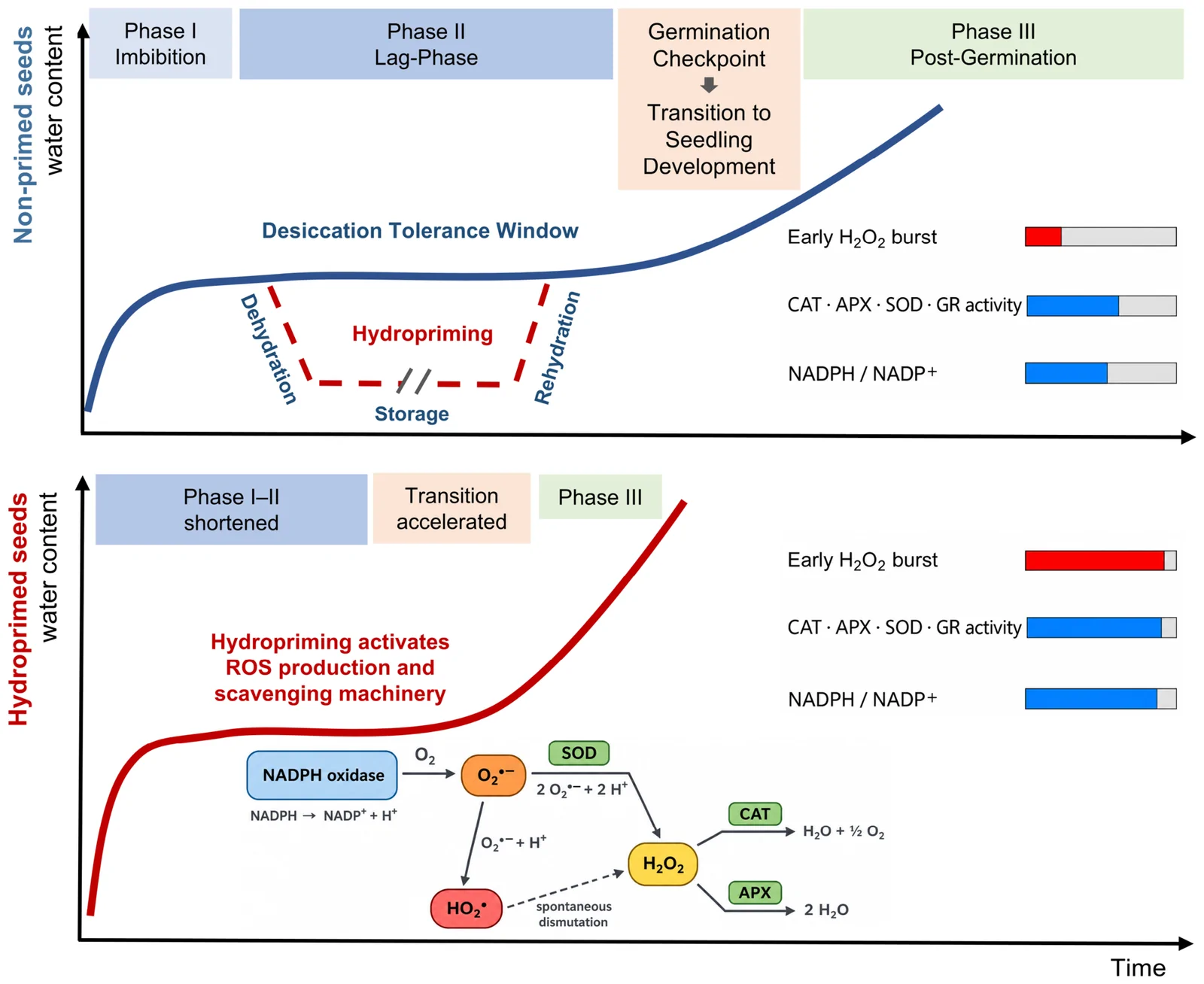
**Comparative analysis of germination phases and ROS metabolism in non-primed vs. hydroprimed seeds. Top Panel**: Triphasic water uptake curve highlights the “desiccation tolerance window” and the germination checkpoint. Red dashed line indicates changes in seed water content during hydropriming. The corresponding bar graphs (right) depict baseline levels of the early H_2_O_2_ burst, antioxidant enzymes activate (catalase, CAT; ascorbate peroxidase, APX; superoxide dismutase, SOD; glutathione reductase, GR) and NADPH/NADP^+^ ratio. **Bottom Panel**: Effects of hydropriming on germination kinetics, illustrating a shortened Phases I–II and accelerated transition to phase III (post-germination). The corresponding bar graphs (right) depict enhanced RBOH level, along with elevated activity of ROS-scavenging enzymes and NADPH/NADP^+^ ratio. **Inserts:** Schematic of the hydropriming-induced ROS signaling pathway, illustrating the role of RBOH in generating superoxide (O_2_^•−^); it is either protonated to hydroperoxyl radical (HO_2_^•^) or dismutated by SOD to hydrogen peroxide (H_2_O_2_). H_2_O_2_ is then can be detoxified by CAT or APX.

The main goal of hydropriming is to provide the embryo with the water required for Phase I and II of germination (germination sensu stricto), but not to allow progression to Phase III, following radicle emergence (Figure 2). Thus, hydropriming includes the three sequential stages [11,12,23].

The first stage is hydration, during which seeds progress through phase I (imbibition) and phase II (lag phase) of germination. The second stage is dehydration involving drying the seeds to their original moisture content. The third stage is rehydration, followed by initiation of embryonic axis growth.

Despite losing water during dehydration, primed seeds do not revert to their pre-treatment metabolic state [11,12,23]. Drying results in delaying germination without compromising viability, leaving the seeds in a quiescent yet metabolically advanced primed state. Crucially, while the hydration and dehydration stages can be cycled repeatedly, the transition to the third stage is irreversible. Rehydration allows seeds to rapidly complete the metabolic cascade. This is likely driven by cellular reprogramming. A highly coordinated sequence of events is triggered, including mitochondrial biogenesis, respiratory activation, DNA and protein repair, reserve mobilization, de novo macromolecular synthesis, and balanced ROS-antioxidant dynamics [10,48,49,50]. As known, hydropriming stimulates macromolecule and cell membrane repair, accelerates germination, promotes uniform seedling emergence, and enhances crop yields [9,10,12,33,51,52]. Although a considerable body of descriptive data confirms the beneficial effects of hydropriming on seed germination and seedling growth, the molecular mechanisms of hydropriming remain poorly understood. In the following sections, we analyze the main metabolic processes occurring in seeds during germination and identify potential targets for hydropriming.

### 2.1. Membrane Resumption and Mitochondrial Biogenesis

In dry seeds, membrane phospholipids are organized in a gel phase with a porous hexagonal structure, resulting in compromised membrane integrity [53,54]. Upon imbibition, this configuration transitions to a lamellar liquid crystalline bilayer, thereby restoring functional membrane structure [54] (Figure 1). This transition is also accompanied by concurrent increases in phospholipid content and unsaturation levels, which point to active membrane lipid remodeling [55]. However, active imbibition can damage membranes, resulting in the leakage of cellular constituents, including inorganic ions (K^+^), amino acids, sugars, organic acids, phenols, and proteins [36,56].

Priming can accelerate the gel-to-liquid-crystalline membrane transition and promote lipid remodeling, thereby reducing imbibitional leakage and preserving cellular constituents [57,58,59]. Posmyk and Janas (2007) reported that hydropriming combined with exogenous proline alleviates chilling-induced membrane lipid peroxidation in mung bean (*Vigna radiata*), supporting the role of priming in preserving membrane integrity under low-temperature stress [57]. Similarly, Ben Youssef et al. (2020) showed that hydropriming and NaCl priming increased membrane lipid content, the phosphatidylcholine/phosphatidylethanolamine ratio, and unsaturation, thereby improving fluidity and reducing damage under salinity [59]. Dhaliwal et al. (2024) showed that hydropriming (8 h at 30 °C) improves membrane integrity and reduces solute leakage under subsequent cold stress [58]. The faster reorganization into a lamellar bilayer significantly reduced electrolyte leakage and preserved cellular constituents, thereby improving germination performance [58].

During the late stage of seed maturation, mitochondria enter metabolic arrest and undergo structural modifications, yet they rapidly regain functionality upon imbibition [60,61]. Consequently, one of the earliest events in imbibition is the progressive proliferation and differentiation of mitochondria [62]. This mitochondrial biogenesis involves the development of structurally simple, quiescent promitochondria, typical of dry seeds, into fully functional cristae-containing mitochondria [60,62,63,64]. The electron transport system is activated immediately upon imbibition. Netzel et al. (2019) directly support this, reporting that O_2_ uptake and ATP accumulation occur within minutes of seed imbibition, indicating rapid activation of mitochondrial respiration [65].

Paszkiewicz et al. (2017) showed that within the first minutes of seed hydration promitochondria gradually acquire a typical shape, reach normal abundance levels, and are capable of generating a membrane potential [61]. By 24 h of germination (lag phase), a fully mature mitochondrial structure becomes visible [34,66]. These structural developments are accompanied by significant functional changes: an increase in mitochondrial protein import, a heightened overall metabolic activity, and a rapid rise in the rate of cellular respiration [34,64,66]. Proteins involved in energy and cell structure in this period are the early translational products of mRNAs stored during late maturation [62,67]. The reactivation of mitochondrial dynamics is positively regulated by gibberellins (GAs) signaling pathway and negatively regulated by abscisic acid (ABA), cold, or inhibited by paclobutrazol treatment [61]. The core findings on O_2_ exchange inside the seeds were recently obtained using microsensors [68]. During seed imbibition, the internal O_2_ content drops to anoxic levels, while the respiration rate increases continuously [68,69]. Minimum O_2_ levels are typically reached in the center of the seed across all major crops, including cereals (e.g., maize, barley, and wheat), legumes (various species), and oilseeds [70]. The primary O_2_ sink is mitochondrial respiration. These mitochondrial events are central to the mechanism of seed priming.

Priming advances mitochondrial biogenesis and the onset of respiration, allowing primed seeds to reach the metabolic and energy status required for germination more rapidly than non-primed seeds [71,72,73]. Mitochondria isolated from primed pea seeds displayed enhanced oxidative properties and respiratory control during early imbibition, directly linking priming with mitochondrial efficiency and ATP production [71,73]. Nie et al. (2020) reported that seed priming significantly enhances the respiration rate and ATP levels in rice seeds and seedlings under chilling stress [72]. The authors attributed these effects to enhanced glycolytic metabolism alongside the repair and biogenesis of mitochondria.

Effective mechanism by which priming increases stress resistance may also involve biomolecular condensates in cytoplasm at the first period of imbibition. Biomolecular condensates are membraneless, organelle-like structures that concentrate molecules, often forming via liquid–liquid phase separation [74]. Recent studies have significantly clarified the molecular mechanisms underlying phase separation in the cytoplasm, wherein proteins and RNAs function as multivalent polymers with adhesive “sticker” motifs [74,75]. These “stickers” engage in weak, non-covalent interactions that, contingent upon specific physicochemical parameters such as temperature, redox state, and pH, drive both intra- and intermolecular associations. Upon crossing a critical system-specific concentration threshold, this process triggers a macroscopic transition into coexisting phases, yielding dense, membraneless organelle-like structures known as *biomolecular condensates* [75]. While the assembly of these condensates is tightly regulated by developmental and environmental cues, their material properties are remarkably dynamic; they can shift from highly fluid, liquid-like states to more solid- or gel-like forms. Crucially, these transitions are of utmost importance for condensate functionality and biological activity [74]. However, recent studies have shown that the formation of biomolecular condensates may be central in the regulation of biochemical reactions in plants and to plant acclimation to stress conditions [74,75,76]. Dorone et al. (2021) demonstrated that FLOE1 allows imbibing seeds to perceive water stress by forming cytoplasmic biomolecular condensates [77]. As an intrinsically disordered, prion-like protein abundant in developing *Arabidopsis thaliana* embryos and seeds, FLOE1 acts as a tunable environmental sensor governing germination via water-potential-dependent phase separation. Under hydrated conditions, it condenses into 1–2 μm droplet-like puncta, upon drying, these structures dissipate into a widespread cytoplasmic distribution. Unlike FLOE1, the nuclear protein SAM8 functions explicitly as a low-water-potential or dehydration sensor [78]. Under normal, water-rich conditions, a negatively charged patch on SAM8 maintains a thick, protective hydration shell that keeps the protein completely diffused.

### 2.2. Thiol Redox System Activation

In the early phases of seed germination, PTM of proteins are recognized as a critical regulatory mechanism [65,79]. Among these, redox regulation centered on the thiol redox state, primarily involving cysteine (Cys) oxidation/reduction, serves as a crucial ‘master switch’ for efficient energy utilization during seed germination [80,81,82]. Within minutes of imbibition, the thiol system shifts from an oxidized (-S-S-) to a reduced state (-SH), leading to activation of the mitochondrial tricarboxylic acid (TCA) cycle and respiratory chain [65,83] (Figure 1).

This thiol-redox machinery is increasingly recognized as a key target through which seed priming exerts its effects [84]. Gerna et al. (2018) showed that hot steam priming advances the onset of seed metabolism, including redox shifts associated with germination [85]. The authors characterized changes in low-molecular-weight thiol-disulfide redox couples (oxidized form of glutathione (GSH)/glutathione (GSH) and cystine/cysteine), demonstrating that priming accelerated the redox transitions required for seed germination and seedling growth [85]. Tripathi et al. (2024) explicitly highlight thioredoxins as key redox-regulated proteins involved in plant priming [84]. It was emphasized that priming plays a vital role in regulating cellular redox homeostasis, with thioredoxins, glutathione, glutaredoxins, and peroxiredoxins acting as central players that mediate these priming effects under stress conditions [84]. Furthermore, Souza et al. (2016) reported that priming improves seed germination and salinity tolerance [86]. This effect correlated with increased expression of the gene encoding thioredoxin (TRX) in seedlings, leading the authors to conclude that the enhanced salt tolerance of primed seeds is directly linked to the upregulation of TRX alongside ascorbate peroxidase (APX), glutathione S-transferase (GST), and the Na^+^/H^+^ antiporter SOS1 (Salt Overly Sensitive 1). Taken together, these findings indicate that priming accelerates metabolic onset and crucial thiol-disulfide shifts while simultaneously upregulating thioredoxin expression and activity to enhance ROS detoxification and stress tolerance in primed seeds and seedlings.

TRX is a small, ubiquitous protein, which contains a redox-active Cys pair within conserved WCGPC active-site motif [87]. Through this pair, TRX catalyzes dithiol–disulfide exchange reactions with target enzymes [87]. The machinery driving thiol-redox changes is entirely dependent on NADPH. This cofactor provides reducing power either to the glutathione/glutaredoxin pathway via glutathione reductases, or to the various TRX via NADPH-dependent thioredoxin reductases (NTR) [83,88]. TRX-dependent reduction in major storage proteins in response to imbibition has been observed in seeds of several species [89,90], an event that affects their solubility and accessibility to proteolysis [91]. TRX-mediated reduction during imbibition has also been documented for non-storage proteins with diverse cellular localizations and functions [90]. This suggests that the reduction in TRX-linked proteins (e.g., TRX h) upon seed rehydration is likely a global and conserved phenomenon in seeds [92]. Spermidine priming of *Allium mongolicum* seeds enhanced the cellular respiration rate and elevated the NADPH/NADP^+^ ratio 1.4-fold [93]. The authors attributed these effects to two main mechanisms: first, the promotion of acetyl-CoA flux into the TCA cycle, and second, the enhancement of NADPH-dependent antioxidant capacity via activation of the pentose phosphate pathway. This elevated NADPH/NADP^+^ ratio provides the essential reducing power for NADPH-dependent thioredoxin reductase to reduce oxidized TRX, thereby maintaining both TCA cycle enzymes and respiratory chain components in their active, reduced states.

Nietzel et al. (2020) focused on the earliest events in energy and redox metabolism that occur in *Arabidopsis* seeds upon imbibition [65]. Using a fluorescent protein biosensing technique for intact seeds, the authors observed rapid ATP accumulation and O_2_ uptake within minutes, providing direct evidence of the rapid activation of mitochondrial respiration. Notably, this activation coincided with a pronounced sharp transition from an oxidizing to a reducing thiol redox state within the mitochondrial matrix. Authors devised a quantitative iodoacetyl tandem mass tag (iodoTMT)-based thiol redox proteomics approach and found that 412 cysteine peptides were redox-switched. These peptides represent central pathways of mitochondrial energy metabolism, including the respiratory chain and every enzymatic step of the TCA cycle. Simultaneously, redox reactions involving reduced glutathione, glutaredoxins, and TRX coordinate with hormones to allow for proper development [65].

### 2.3. Repair of Nucleic Acids and Proteins

To mitigate damage sustained under water loss at the late maturation, during stor-age, and, most critically, imbibition, seeds deploy a coordinated suite of repair mechanisms (Figure 1). This response encompasses the restoration of compromised cellular membranes, the refolding of proteins, and the repair of damaged nucleic acids [28,34,64]. Repair is triggered when hydrated tissues reactivate enzymatic systems and boost mitochondrial energy production [35,65]. To support this process, seeds program repair by up-regulating genes that encoded the relevant long-lived mRNAs during late maturation. This ensures the rapid translation of required enzymes during the imbibition stage, al-lowing for swift damage repair [33,65,66].

Repair systems play a central role in seed priming, a controlled hydration treatment that allows seeds to progress partway through imbibition before being dried back, providing a critical window for the translation and activation of repair enzymes encoded by long-lived mRNAs. Consequently, primed seeds enter subsequent imbibition with a partially “pre-run” repair machinery. Gran et al. (2025) used polysome profiling and ribosome nascent chain complex sequencing (RNC-seq) to show that hydroprimed seeds contain more mRNA–ribosome complexes than mature dry seeds, demonstrating that germination-essential mRNAs are already docked with ribosomes for accelerated protein synthesis upon re-imbibition [94]. Waterworth et al. (2025) found that priming robustly activates the transcriptional response to chromosomal breaks, proving through Arabidopsis mutants that DNA double-strand break repair is a critical determinant of primed-seed longevity [95]. Together, these findings confirm that controlled hydration (that is, seed priming) structurally and transcriptionally primes both the translational apparatus and DNA repair systems to optimize germination readiness.

DNA damage must be repaired promptly during early imbibition, prior to the initiation of the cell cycle (and subsequent cell division), to maintain germination potential and minimize growth inhibition and mutagenesis in subsequent seedling development [67]. Of particular concern are chromosomal breaks, or DNA double-strand breaks, which are highly cytotoxic. They can lead to chromosome fragmentation, loss of genetic information, and cell death [68]. DNA repair in seeds employs universal eukaryotic mechanisms: homologous recombination (HR) and non-homologous end-joining (NHEJ) to fix double-strand breaks, plus nucleotide excision repair (NER), base excision repair (BER), and correction of unpaired bases [65,69,70]. Oxidative damage of seed DNA is mostly associated with ROS-mediated modifications, including guanine oxidation to 8-oxo-2′-deoxyguanosine, thymine oxidation to thymine glycol, and oxidative cleavage of the sugar-phosphate backbone leading to strand breaks [66,71]. The major oxidative modification of seed DNA, as shown in Arabidopsis, is the hydroxylation of guanine, which yields the potentially mutagenic base 7,8-dihydro-8-oxoguanine (8-oxoG) [72]. This modified base can form complementary pairs with both cytosine and adenine, which may result in errors during DNA replication [65,73]. The removal of 8-oxoG from the DNA double helix typically relies on the BER system [72]. The entire process proceeds in several stages. First, the damaged base is recognized and removed by cleavage of the corresponding N-glycosidic bond, catalyzed by a DNA glycosylase [73]. This reaction generates apurinic/apyrimidinic (AP) sites, which serve as substrates for 8-oxoguanine DNA glycosylase/lyase (OGG). This enzyme cleaves the sugar-phosphate backbone of the DNA, creating a single-strand break. Subsequently, DNA polymerase beta (POLB) initiates repair of the DNA chain [72]. Thus, the BER pathway encompasses several key steps: the excision of the damaged DNA base, cleavage of the sugar-phosphate backbone at AP sites, clean-up of the resulting DNA ends, gap filling through DNA synthesis, and, finally, DNA ligation [73]. In addition to other types of DNA damage, double-strand breaks require dedicated repair mechanisms [65]. DNA glycosylases responsible for repairing base lesions include key enzymes such as OGG and formamidopyrimidine-DNA glycosylase (FPG) [72,74]. Once these glycosylases cleave the sugar-phosphate backbone, DNA ligases seal the resulting breaks to complete the repair process [65,75].

Kiran et al. (2020) found that treatment of *Solanum melongena* seeds with 320 mM NaCl enhanced germination by reactivating DNA repair pathways [76]. This reactivation included the OGG1 upregulation, as well as the activation of NHEJ and HR [76]. Another crucial player in DNA repair, particularly for damage related to dehydration, is poly(ADP-ribose) polymerase (PARP). PARP catalyzes poly-ADP-ribosylation, a process that reversibly modifies proteins by attaching a chain of ADP-ribose units [77]. Notably, the activity of PARP is directly stimulated by the presence of DNA strand breaks, linking it closely to the detection and repair of DNA damage.

Seed priming triggers the upregulation of vital DNA repair machinery, including the BER, NHEJ, and HR pathways, as well as elevated PARP activity, which collectively serve as the underlying molecular mechanisms preserving genomic integrity during early germination. A comprehensive review by Sincinelli et al. (2025) consolidates evidence across multiple species, highlighting the specific role of the OGG1 enzyme within these pathways [96]. Further supporting this, Waterworth et al. (2025) demonstrated that priming directly activates the DNA damage response in Arabidopsis seeds [95]. Specifically, their study revealed a significant elevation in the expression of the top four most inducible DNA repair genes: *RAD51*, *XRI1*, *PARP2*, and *BRCA1*. Once of targets underlying the reparation is stored messenger RNAs (mRNAs), which are highly susceptible to oxidative damage by ROS [79,80,81]. These mRNAs, synthesized during late seed maturation and translated upon germination, are essential for initiating seedling growth. Their successful translation depends on the preservation of length to maintain an intact coding sequence, secondary structure for ribosome interaction, and specific motifs such as the 5′ cap and poly-A tail [75]. Their reparation comprises ATP-dependent RNA ligases which rejoin broken RNA strands, nucleotidyl transferases, and modifying enzymes such as phosphatases and kinases which prepare RNA ends for ligation, and methylases which add protective methyl groups to RNA termini [66,82].

A key element of protein protection in seeds is the reversible formation of disulfide bonds (S–S). In their reduced sulfhydryl (SH) form, proteins are initially synthesized but are subsequently oxidized to yield a more stable disulfide configuration [83]. In dry seeds, this structural change makes proteins more compact, less susceptible to degradation, and protects them against pro-teases [84]. Upon germination, the original sulfhydryl form is restored by thioredoxins, enabling the reactivation of protein functions [63]. In dry seeds proteins can accumulate various spontaneous, non-enzymatic post-translational modifications, including oxidative, glycation, and lipoxidation events [85,86,87,88]. While most of these modifications are irreversible, some oxidative changes at specific amino acid residues can be reversed through both enzymatic and non-enzymatic mechanisms [89]. One such repair mechanism involves methionine sulfoxide reductase (MSR), which reduces methionine sulfoxide back to methionine [84]. The loss of protein function can also result from the conversion of aspartate or asparagine residues into abnormal isoaspartic acid by spontaneous covalent modification [90]. This damage is repaired in plants by L-isoaspartyl methyltransferase (PIMT), which methylates damaged L-isoaspartyl and D-aspartyl residues via their side-chain carboxylic groups [90]. PIMT also facilitates the hydrolysis of L-succinimide—an intermediate formed during non-enzymatic rearrangement—restoring normal L-amino acid residues. Experimental evidence shows that overexpression of PIMT1 in Arabidopsis enhances seed longevity, whereas reduced PIMT1 expression leads to a loss of seed vigor [91,92]. Although PIMT induction by ABA is well-documented, the precise molecular mechanisms driving this regulation have long remained elusive. Kamble et al. (2022) demonstrated that ABSCISIC ACID INSENSITIVE4 (ABI4) directly binds to the PIMT1 promoter to regulate its expression in Arabidopsis seeds [93]. The authors propose that AtABI4 plays a central role in modulating AtPIMT1 expression, thereby imparting seed vigor and longevity to orthodox seeds.

Priming appears to support RNA repair and the protection of stored mRNAs, and to favor the maintenance of redox-regulated protein protection mechanisms, including the reversible formation of disulfide bonds and the activity of MSR and PIMT [94,97].

## 3. Key Regulatory Pathways Associated with Seed Priming

### 3.1. Hormonal Regulation

The use of various phytohormones as priming agents has become a widely accepted approach with proven positive effects, especially in enhancing plant stress resistance [10,98,99]. Currently, hormonal seed priming is a commonly used technique to improve seed germination, seedling growth, and crop yield in adverse conditions. In hormonal seed priming, seeds are pre-soaked in media with phytohormones. This treatment enhances germination, seedling growth, and crop yield by improving nutrient uptake, stimulating root development, and boosting overall physiological activity [22,99]. Rhaman et al. (2020) provide a comprehensive review of the effective approaches of using phytohormones for seed priming to mitigate abiotic stress [98].

The transition from seeds to seedlings is strictly controlled at the genetic level with the ABA-dependent transcription factors. Among them, master regulators comprises LEAFY COTYLEDON 1 (LEC1), ABSCISIC ACID INSENSITIVE3 (ABI3), FUSCA3 (FUS3), and LEC2 (known as a LAFL network), along with DELAY OF GERMINATION 1 (DOG1), ABI4 and ABI5 [39,100,101,102,103,104]. Activated by elevated ABA level, these proteins control the expression of genes that govern critical seed-specific traits, including maturation, dormancy, and germination [105,106,107]. As known, ABA promotes dormancy, while GAs induce dormancy release and germination [1,48,106,108,109,110,111,112,113,114,115,116,117]. The increase in GAs results in degradation of DELLA proteins and a subsequent decrease in ABA content [3]. Notably, it was shown that ABA level may increase during imbibition at stress condition [118]. ABA was found to be produced and released by the endosperm, transported to the embryo, where it represses radicle extension and subsequent embryonic growth under unfavourable conditions [119,120]. This process requires *RGA-Like2 (RGL2)* expression, which is a negative regulator of GAs signaling [3,121].

Regulation of seed germination extends beyond ABA and GAs alone, involving intricate cross-talk with other signaling pathways [3,117,122]. Other hormones, such as auxins [112,122,123,124], brassinosteroids (BRs) [122,125], ethylene [126,127,128], cytokinins [122,129], and jasmonates [130], also contribute to this regulation. Auxin has dual, dose-dependent effects on seed germination [131]. High auxin level promote seed dormancy by activating *ABI3* through a pathway mediated by AUXIN RESPONSE FACTOR10 (ARF10) and auxin signaling repressor (IAA8) [123,132]. At low concentrations, auxin acts in concert with GAs to stimulate embryo cell growth. BRs, like GAs, promote seed coat rupture to stimulate *Arabidopsis* germination. Moreover, germination does not appear to depend entirely on GAs alone but rather on GAs and BRs working in concert [3,133]. Ethylene is another promoter of germination [126,134,135]. Ethylene stimulates root formation, germination, and seedling establishment by influencing and interacting with GAs biosynthesis [126,127,128]. On the other hand, ethylene counteracts the effects of ABA by regulating ABA metabolism genes and signaling pathways [127].

### 3.2. ROS as a Regulator of Seed Germination

ROS are considered as a key element of plant signaling that controls cell growth, tissue development, and responses to environmental stresses [136,137,138,139,140]. The major sources of ROS are electron transporting chains in mitochondria and chloroplasts, NADPH oxidases (NOX), also known as RBOH (Respiratory Burst Oxidases), in the plasma membrane, cell wall peroxidases and oxidases as well as several other systems, such as xanthine oxidase and glucose oxidase [137,141]. The most important enzymes producing ROS de novo for regulatory and developmental needs are NOX [137,142,143,144].

In 2007, Oracz et al. demonstrated that ROS triggered protein carbonylation to release dormancy [145]. Thereafter, extensive further evidence has shown that ROS balance is critical for germination [37,140,146,147]. Extremes in ROS levels can impair embryo viability; therefore, ROS in germinating seeds needs to be tightly controlled [148]. This concept of an “*oxidative window*” highlights the dual role of ROS that can both promote and inhibit seed germination [148,149]. Undoubtedly, NOX and peroxidases will also play a significant role in redox-regulated responses in seeds. Some authors suggest that the mitochondrial source of ROS is the most significant, although this requires more experimental evidence [65,150].

Even when environmental conditions are favorable, ROS levels in seeds are maintained at a level sufficient to trigger hormonal signaling [151]. ROS oxidizes negative regulators of germination, leading to activation of GAs signaling and inactivation of ABA signaling [37,146]. Specifically, H_2_O_2_ alters the ABA/GAs balance by promoting the expression of *CYP707A2* genes (involved in ABA degradation) and increasing the expression of *GA3ox1* genes (involved in GA biosynthesis) [152,153]. In barley (*Hordeum vulgare*) aleurone cells the production of H_2_O_2_ was induced by exogenous H_2_O_2_ with following induction of *α*-amylases [154].

ROS are also crucial for the weakening of cell walls in the seed coat and micropylar endosperm, which enables the radicle to emerge [155,156,157,158]. The direct in vivo evidence for hydroxyl radical-mediated cell wall loosening during plant seed germination and seedling growth was first obtained for *Lepidium sativum* seeds [159]. ROS-dependent endosperm weakening preceded radicle emergence in this species. This process is mechanistically similar to the cell wall softening that occurs during growth by cell expansion [155,156,157,158]. A significant ROS generation was detected in the elongation zone of radicles in germinating pea seeds [160]. The main source of the oxidative burst accompanying radicle elongation is the O_2_^•−^ generated by NOX [143,155,161,162]. NOX, together with apoplastic copper-containing amine oxidases and polyamine oxidases, is hypothesized to function in the late events of seed germination by generating ROS within the radicle cell wall and facilitating the cleavage of seed coat polysaccharides [161,163]. A fluorescent probe tests was shown positive results for ROS in root tips of germinating seeds of cress [159] and willow [164]. Consequently, ROS, such as H_2_O_2_ has emerged as a common tool for seed priming [10,165,166,167,168,169,170].

Bykova and Igamberdiev (2025) demonstrated that the enhanced accumulation of nitric oxide (NO) and ROS is critical for inducing germination [79]. Beyond germination, ROS and NO also serve as key mediators of seed priming [171,172,173] (Figure 2). Ren et al. (2023) investigated the role of ROS in hydropriming by comparing it with osmopriming and examining its subsequent effects on seed deterioration [171]. The authors demonstrated that priming modulates the deterioration rate of rice seeds by regulating ROS accumulation, seed respiration, and starch degradation [171]. Crucially, their study provides direct evidence that hydropriming accelerates ROS generation, indicating that the hydropriming-induced ROS burst is a central, measurable physiological event. Complementing these findings, Pagano et al. (2023) provided a comprehensive analysis of the molecular mechanisms underlying seed priming, focusing specifically on the central role of ROS [10]. The authors detailed the “dual role” of ROS in early seed germination—a concept fundamental to understanding priming efficacy. They explained that while ROS generated during seed rehydration can cause cellular damage, successful germination depends on an “oxidative window” in which ROS act as signaling molecules to trigger the process. Furthermore, the study highlights how the balance between ROS accumulation and the seed’s antioxidant response serves as a key indicator of seed quality, which is directly modulated by priming techniques.

A review by Fejes et al. (2025) highlights the integral role of NO in priming-induced tolerance [173]. NO is a gaseous free radical that readily diffuses across the plasma membrane, making it an ideal autocrine (intracellular) and paracrine (intercellular) signaling molecule [174]. NO sensitivity is an ancient trait mediated by hemoproteins containing Heme-Nitric Oxide/Xygen (H-NOX) binding domains (IPR038158). While these domains function as NO sensors in bacteria, fungi, and animals (including humans), they were long thought to be absent from plants. Recently, however, several NO-sensitive hemoproteins with conserved H-NOX domains have been discovered in plants [175]. As a second messenger, NO plays a crucial role in plant growth, development, and adaptation to both biotic and abiotic stresses [174]. NO is essential for regulating seed dormancy and germination, primary root growth and adventitious root development, pollen tube growth, anthesis initiation and leaf senescence, stomatal movement, and responses to salinity, extreme temperatures, high light, and pathogen attack [176]. It was reported that various types of seed priming, including osmotic, chemical, physical, hormonal, and redox treatments elevate endogenous NO levels, thereby establishing it as a key mediator of growth regulation and defense responses [173]. Concurrently, the exogenous application of the NO donor sodium nitroprusside during priming effectively mitigates abiotic stresses such as salinity, cold, drought, and heavy metal toxicity.

## 4. Epigenetic Mechanisms of Germination in the Context of Stress Memory

To enhance survival, plants have evolved adaptive mechanisms that enable them to “remember” prior stress exposures and mount faster, stronger responses upon recurrence—a phenomenon termed stress memory [47,177,178,179]. Prior stress can leave molecular, metabolic, and physiological imprints that alter the magnitude, speed, and quality of subsequent responses, enabling adaptive plasticity to challenge such as drought, heat, or pathogen attack. This memory is not a transient physiological adjustment, but a regulated, semi-stable state associated with epigenetic regulation [178,179,180,181,182]. Like other eukaryotes, plants possess a highly complex set of non-coding and epigenetic mechanisms that regulate gene expression [183,184,185,186]. The pivotal components of these pathways during seed germination include DNA methylation, histone post-translational modifications, and chromatin remodeling, in addition to non-coding RNAs that direct the silencing machinery to specific genomic regions [127,128,129,130,131]. Together, these epigenetic modifications form a robust, multifactorial foundation that regulates plant development and environmental adaptation [187,188,189,190,191]. MacDonald and Mohan (2025) demonstrated that the physiological benefits of chemical seed priming agents (salicylic acid, zinc, gibberellic acid, and potassium nitrate) are inherited across generations via epigenetic pathways, as evidenced by distinct alterations in DNA methylation and histone modification [192].

*DNA methylation* is a foundational epigenetic mechanism driving seed priming effects, particularly in establishing plant stress memory, enabling the transgenerational inheritance of enhanced stress tolerance, and regulating gene expression changes that persist well beyond the initial treatment [193,194]. Both seed development and germination are accompanied by dynamic reprogramming of DNA methylation profiles, mediated by two distinct processes: maintenance methylation and de novo methylation [195]. Consistent with these findings, recent reviews identify a broader suite of epigenetic mechanisms, including DNA methylation, histone modifications, and nucleosome positioning—as the core molecular drivers of priming and stress memory [196,197,198,199]. These studies focused on the retention of induced epigenetic marks and concluded that seed priming can effectively fortify and sustain stress tolerance in subsequent generations. Notably, Auler and do Amaral (2025) emphasized the precise role of DNA methylation in this transgenerational inheritance, highlighting its direct practical applications in agronomic techniques such as seed priming and hardening [197].

In general, DNA methylation involves the covalent modification of cytosine residues. In contrast to most other organisms, in which this modification occurs almost exclusively within cytosine–guanine (CG) dinucleotide contexts, plants exhibit DNA methylation at CG, CHG, and CHH sequences, where H denotes A, T, or C [193,194]. Within coding sequences, methylation is predominantly found at CG sites, whereas non-CG methylation is far less frequent [200]. Indeed, CG methylation accounts for over 50% of total cytosine methylation in angiosperms [201]. Beyond these context-specific differences, overall genomic methylation profiles vary widely across plant species, with CHG and CHH contexts showing markedly higher heterogeneity than CG sites [201,202,203]. Several distinct DNA methyltransferases mediate both the de novo generation and subsequent maintenance of DNA methylation across the three sequence contexts. In *Arabidopsis*, DNA METHYLTRANSFERASE1 (MET1) is the primary enzyme responsible for maintaining CG methylation [204,205]. In contrast, methylation at CHH and CHG sites generally depends on two enzymatic systems: CHROMOMETHYLASE2/3 (CMT2/CMT3) and DRM2 [206,207,208]. DNA methylation patterns continuously change during development [186,209,210,211]. The occupancy of CHH methylation sites increases remarkably from the early to late stages of seed development and gradually decreases later during germination. Specifically, both RNA-directed DNA methylation (RdDM) and CMT2 are responsible for CHH methylation in developing seeds but lose their activity upon germination [193]. In contrast to CHH sites, the patterns of CG and CHG methylation remain, to a large extent, unchanged over the entire period of seed development [193,209,210,212].

Multiple genes involved in seed development and germination reside within hypomethylated genomic regions termed *DNA methylation valleys* (DMVs) [213]. The methylation status of these regions remains unchanged throughout seed development, from fertilization to germination. For instance, genes encoding GA-biosynthesis enzymes (such as *GmGA20Ox2*, *GmGA3Ox1*, *AtGA20Ox2*, and *AtGA3Ox1*) and various transcriptional regulators are all located within these hypomethylated regions in both soybean and *Arabidopsis* [213]. Plant DNA demethylation proceeds via either passive or active mechanisms. Passive demethylation arises when the newly synthesized DNA strand fails to undergo methylation during replication, so that only the parental strand remains methylated [214].

*Histone modifications* play a crucial role in regulating gene expression networks that control seed development, dormancy, and the transition to germination in response to environmental signals. Different types of histone PTMs have been described to date in the context of the epigenetic regulation of seed development and germination [183]. Thus, alterations in chromatin structure leading to gene expression changes can be mediated, at least in part, by acetylation, methylation, phosphorylation, ubiquitination, and SUMOylation of histones [183]. These modifications play an important role in the control of seed maturation, dormancy, and germination [101,215]. Thereby, the patterns of histone modifications collectively referred to as the “histone code” serve as docking marks for the recruitment of chromatin-remodeling proteins and other transcriptional regulators [216].

Acetylation and methylation of lysine residues in histone H3 directly affect the expression of associated genes. The cycles of histone acetylation and deacetylation, catalyzed by histone acetyltransferases and deacetylases, respectively, are critical elements in the regulation of genome activity [217,218]. The acetylation of lysine side chains neutralizes the overall positive charge of histones and alters the surface charge distribution [219,220], resulting in chromatin decondensation and associated with higher transcriptional activity. Conversely, this relaxation can be reversed by the activity of histone deacetylases, which restore the compact chromatin conformation [221].

Histone methylation at lysine (K) and arginine residues is a versatile regulatory mechanism that modulates protein–protein interactions [222]. Depending on the specific residue and its methylation state (mono-, di-, or tri-methylation), this post-translational modification can drive either transcriptional activation or repression [222]. These opposing processes are mediated by the Trithorax (TrxG) and Polycomb (PcG) complexes which activate and repress gene transcription, respectively [223].

In *Arabidopsis thaliana*, trimethylation of histone H3 at lysine 4 (H3K4me3) serves as a canonical hallmark of active transcription and is essential for the precise temporal regulation of seed maturation [224]. Deposition of this activating mark is catalyzed primarily by the *Arabidopsis* TrxG and TrxG-related proteins, which act as dedicated H3K4-specific methyltransferases [224]. Conversely, the removal of H3K4me3 is mediated by Jumonji C (JMJ) domain-containing demethylases, ensuring a dynamic equilibrium of this mark throughout seed development [224,225].

Beyond H3K4me3, other methylation marks play critical roles in these processes. For instance, H3K27me3, which is associated with gene repression and deposited by the Polycomb Repressive Complexes 1 and 2 (PRC1 and PRC2) also critically contribute to the maintenance of dormancy and the repression of germination-promoting genes [226,227,228]. Polycomb group proteins form chromatin-modifying complexes that mediate transcriptional silencing, enabling the coordinated repression of genes [229,230,231]. During seed germination, PRC1 functions as a critical epigenetic switch that enables the transition to vegetative growth by silencing maturation genes, including the LAFL network and DOG1 [232]. At the physiological level, this chromatin repression deactivates the ABA pathway responsible for maintaining seed dormancy. Consequently, the inhibition of GAs biosynthesis and signaling is lifted, driving downstream developmental processes such as cell elongation and radicle emergence. Recent findings demonstrate that H3K4me3 directly antagonizes PRC2 by competing at the EED/FIE binding site, thereby blocking H3K27me3 spreading and reinforcing active transcription boundaries [227,233,234].

*Noncoding RNAs*: Plant genomes harbor not only protein-coding genes but also diverse non-coding sequences, including regulatory non-coding RNAs (ncRNAs) [185,235,236]. Key classes of ncRNAs, such as microRNAs (miRNAs), long non-coding RNAs (lncRNAs), small interfering RNAs (siRNAs), and circular RNAs (circRNAs) play crucial roles in gene regulation. LncRNAs modulate transcription by interacting with chromatin and transcriptional regulators, whereas miRNAs exert post-transcriptional control through targeted mRNA cleavage and degradation [185]. sRNAs are central to the regulation of gene expression throughout the plant life cycle, orchestrating the expression of target genes from embryogenesis through the establishment of vegetative identity to ensure smooth developmental phase transitions [211]. Among sRNAs, miRNAs, a major class of 20–24 nt ncRNAs, post-transcriptionally regulate key processes such as mRNA cleavage, translational repression, and DNA methylation [235,237,238,239]. Notably, miRNAs play critical roles in multiple processes, seed germination [211,240].

Seed germination is influenced by miR159 and miR160, which target transcripts of the myeloblastosis family members MYB33, MYB65, and MYB101, and ARF10, respectively [240,241]. Alterations in the abundance of these miRNAs, or in the sensitivity of their target transcripts, can modify the germinating seed’s response to the suppression of ABA biosynthesis [240,241]. Additionally, five other miRNAs: ath-miR8176, ath-miR851-5p, ath-miR861-3p, ath-miR158a-5p, and ath-miR779.2 were identified as exhibiting peak expression during germination. Among these, miR851 is predicted to target the pentatricopeptide repeat (PPR) gene family, whose transcripts are also upregulated during this stage. Finally, because several predicted targets of miR858a (MYB13, MYB65, and MYB93) are known regulators of germination, this miRNA may also contribute to the process [242]. LncRNAs are transcripts longer than 200 nucleotides that lack protein-coding potential but otherwise resemble mRNAs, featuring a 5′ cap, spliced introns, a polyadenylated tail, and transcription via RNA polymerase II [185]. Although eukaryotic genomes such as that of *Arabidopsis* contain thousands to tens of thousands of lncRNAs, only a small fraction have been experimentally characterized as critical regulators of plant development and stress responses via chromatin and protein interactions [243]. A prominent example is the regulation of the *DOG1* [244]. Fedak et al. (2016) demonstrated that an autonomous promoter in the 3′ region of *DOG1* drives the expression of a distinct antisense noncoding RNA (*asDOG1*), which strongly suppresses *DOG1* expression during seed maturation [244].

*Chromatin remodeling* is a critical ATP-dependent epigenetic mechanism that dynamically orchestrates genome accessibility [245]. They do not chemically modify DNA or histones but physically reorganize nucleosomal architecture. By mobilizing, ejecting, or restructuring nucleosomes, this process enables transcription factors and the transcriptional machinery to access otherwise tightly packaged DNA, thereby precisely modulating gene expression. The physical reorganization of chromatin is primarily executed by multisubunit ATP-dependent remodeling complexes, all of which contain a conserved SNF2-type ATPase subunit characterized by DExx and HELICc helicase-like domains [246,247]. Based on their architecture and mechanisms, these complexes are grouped into major families: SWI/SNF, ISWI, CHD, and INO80, that perform diverse nucleosomal tasks, including histone sliding, eviction, canonical-to-variant histone exchange, nucleosome maturation, and regulation of internucleosomal spacing [246,247]. Among these, Switch deficient SUCROSE NONFERMENTING (SWI/SNF) complexes exhibit a unique functional duality: they can both slide the histone octamer along the DNA and completely evict it, profoundly altering chromatin states. Consequently, SWI/SNF remodelers are indispensable for cell fate reprogramming, often acting in coordination with pioneer and non-pioneer transcription factors to reshape the regulatory landscape [248].

Simon et al. (2026) show that dry Arabidopsis embryos accumulate the histone variant H2B.8, which organizes higher-order chromatin via spatial clusters and heterotypic nucleosomes at euchromatic transposons and lowly expressed genes [249]. During imbibition, H2B.8 modulates their transcriptional activation. Subsequent water uptake reduces H2B.8 transcripts and evicts the protein from chromatin independently of DNA replication but dependent on de novo transcription. Imbibition also induces H3.3 turnover, revealing a replication-independent chromatin reprogramming essential for early plant development [249]. Critically, chromatin remodeling functions in concert with other epigenetic layers, including DNA methylation and histone PTM [247,250], and plays a central role in plant hormone signaling and stress responses [251].

To summarize, epigenetic signals including dynamic changes in DNA methylation, demethylation, histone post-translational modifications, and non-coding RNA-related and chromatin remodeling regulatory networks act as master modulators of seed development and the critical transition from seed to seedling. While the roles of reversible DNA methylation and histone modifications during germination are well-established, many specific miRNAs and their precise target genes remain largely uncharacterized. These reversible modifications can serve as highly effective potential targets for various seed priming techniques, allowing for the precise manipulation of gene expression to enhance germination uniformity, seedling vigor, and stress tolerance. However, beyond their fundamental regulatory functions, these epigenetic modifications exhibit inherent plasticity and reversibility, which makes them exceptionally attractive and effective potential targets for various seed ‘primeomics’ techniques [47]. An increasing body of evidence indicates that diverse priming protocols exert their beneficial effects on germination uniformity, speed, and stress resilience, at least partially, through the targeted reprogramming of the seed’s epigenome [180,181,192]. This reprogramming involves the erasure of inhibitory methylation marks at growth-promoting loci, the modulation of histone acetylation states to establish a transcriptionally permissive chromatin architecture, and the alteration of small RNA populations [181]. These findings indicate that epigenetic marks can be targeted during priming, presenting critical biotechnological opportunities for engineering climate-resilient crops.

Consequently, seed stress memory represents a paradigm shift in our understanding of plant adaptation. It reveals seeds not as passive propagules but as dynamic biological systems capable of retaining and utilizing molecular signatures of prior environmental stresses [182,252,253]. Priming acts as a practical means to induce this “primed” physiological state, thereby enhancing germination, vigor, and stress tolerance. As Srivastava et al. (2021) note, stress memory and seed priming are essentially two faces of the same coin [47]. By harnessing this memory through priming techniques whether via mild primary stress or biological and chemical agents, researchers can establish stress memory and promote more robust adaptation at later developmental stages. This strategy holds considerable promise for developing climate-resilient crops [252]. Priming-induced stress memory does not rely on a single pathway; rather, it emerges from a highly complex signaling network. Unravelling this network transform precision agriculture, by providing a framework to customize priming protocols and strengthen crop resilience against in the face of increasingly unpredictable climatic conditions.

## 5. Conclusions

Seed germination and early seedling establishment are critical checkpoints in the plant life cycle, during which plants are exceptionally vulnerable to diverse environmental and biotic stresses. During this stage of ontogenesis, seedlings lack fully developed protective structures and systemic defenses, rendering them highly susceptible to abiotic constraints. Failure to establish successfully at this stage can reduce stand uniformity, compromise subsequent growth, and limit crop productivity. To mitigate these challenges, seed priming has emerged as a highly efficient approach in modern agriculture, leveraging mechanisms that parallel natural stress-adaptation phenomena. A growing body of scientific literature underscores seed priming as a cornerstone of both fundamental and applied research in seed biology, plant breeding, and biotechnology.

However, the molecular mechanisms underlying seed priming remain poorly understood. It remains unclear how the intricate interplay among ROS, hormonal signaling networks (ABA, GAs, and others), and redox-sensitive regulatory proteins orchestrates the transition from dormancy to germination and enhances stress tolerance in primed seeds. Similarly, the precise signal transduction pathways through which germinating seeds perceive and respond to fluctuations in water potential have yet to be clearly elucidated.

Given the demand to optimize crop performance under increasingly unpredictable climatic conditions, future research must shift from empirical optimization toward a comprehensive, mechanistic understanding of priming techniques. A key priority is to identify the specific cellular and molecular processes modulated by priming, including mitochondrial biogenesis, chromatin remodeling, DNA and protein repair machinery, de novo macromolecular synthesis, and ROS/redox homeostasis. Furthermore, the epigenetic reprogramming associated with priming, particularly the dynamic regulation of DNA methylation and histone modifications, offers a promising avenue for understanding how environmental cues are integrated into long-lasting adaptive responses.

While different types of priming are demonstrably valuable for agricultural practice, their underlying mechanisms can now be comprehensively decoded using modern multi-omics approaches, real-time biosensors, and advanced genetic tools. Such mechanistic insights will not only illuminate fundamental aspects of seed biology but also facilitate the rational optimization of priming methodology. Ultimately, elucidating the molecular basis of priming tools will empower scientists and farmers to deploy highly effective priming strategies, thereby enhancing crop resilience, ensuring food security, and advancing agricultural sustainability.

## Data Availability

No new data were created or analyzed in this study. Data sharing is not applicable to this article.

## Abbreviations

The following abbreviations are used in this manuscript:

ABA	Abscisic acid
ABI	ABA-INSENSITIVE
APX	Ascorbate peroxidase
ARF	AUXIN RESPONSE FACTOR
BER	Base excision repair
CAT	Catalase
CMT	CHROMOMETHYLASE
DOG1	DELAY OF GERMINATION1
FUS	FUSCA
GAs	Gibberellic acids
GR	Glutathione reductase
GSH	Glutathione
GSSG	Oxidized form of glutathione
LAFL	Network of transcription factors (LEC1, ABI3, FUS3 and LEC2)
LEC	LEAFY COTYLEDON
ncRNAs	Non-coding RNAs
NER	Nucleotide excision repair
NHEJ	Non-homologous end joining
NOXs	NADPH oxidases
NTR	NADPH-dependent thioredoxin reductase
PTM	Post-translational modifications
RBPs	RNA-binding proteins
RdDM	RNA-directed DNA methylation
ROS	Reactive oxygen species
SOD	Superoxide dismutase
TCA	Tricarboxylic acid
TRX	Thioredoxin
